# Effect of Relative Density on Compressive Load Response of Crumpled Aluminium Foil Mesh

**DOI:** 10.3390/ma12234018

**Published:** 2019-12-03

**Authors:** David Hughes, Emeka H. Amalu, Tannaz Pak, Ryan Kennedy

**Affiliations:** Department of Engineering, School of Science, Engineering and Design, Teesside University, Middlesbrough TS1 3BA, UK; e.amalu@tees.ac.uk (E.H.A.); t.pak@tees.ac.uk (T.P.)

**Keywords:** crumpled aluminium foil mesh (CAFM), compression, effective yield strength, relative density, expanded metal

## Abstract

In recent years, a large number of metal foams and porous metals have been developed. Due to the high cost of these materials alternative manufacturing methods for cellular metallic materials are being explored. Crumpled metallic foil meshes, manufactured via die compression techniques, are evolving as a potential alternative method. However, the non-availability of sufficient data on their load response is limiting their uptake. Uniaxial compressive load response of crumpled aluminium foil meshes (CAFMs) of varying densities, forged by open and closed die compression, are studied. A 0.05 mm thick aluminium sheet mesh, manufactured by the expanded metal process is used. X-ray computed micro-tomography is employed to image the CAFM’s internal cellular structure. The stress-strain relation demonstrates that the CAFMs produce identical load response profile irrespective of their relative density. Power law functions $E_{R}=17110\rho_{r}{}^{3.6547}$ and $\sigma_{Y,E}=53.092\rho_{r}{}^{2.2249}$ define the relationships between real Young’s Modulus $E_{R}$ and effective yield strength, $\sigma_{Y,E}$. The study provides new knowledge on the effect of relative density on the compressive properties of CAFMs which have applications across lightweight structural design.

## 1. Introduction

The use of cellular materials in the design and fabrication of lightweight, high-value, engineered structures is evolving owing to their impressive mechanical properties and because they weigh a fraction of the solid material they are made of [1]. They are used in a wide variety of applications ranging from commercial to military, including automotive, aerospace and construction sectors [2,3]. Cellular materials achieve low weight because they are made up of an interconnected network of struts, resulting in a porous structure [4]. Cellular materials in general have been the focal point of many facets of research in recent decades, especially for uses as core materials for composite sandwich panels [5]. A series of reviews of cellular materials and their applications have been published [6,7,8]. Recent works concentrate on biomimetics [9], honeycomb structures [10] and entangled materials [11,12].

High porosity metals are separated into two distinct categories, those with closed pores usually referred to as foams and those with open, connected cells referred to as porous metals [13]. The expanded metal foil mesh utilised in this study, once compacted, forms a permeable structure comparable to porous metal structures. Traditional methods for creating porous metal structures include electroplating polymer foam templates, sintering and selective laser sintering [6,7]. While effective, these methods are all complex and expensive limiting the applications of porous metal media [14,15,16,17]. The significance of developing alternative processes for low-cost, light-weight, metal foams has been discussed and evaluated by Lehmhus [18].

Limited work has been carried out on crumpled sheet materials. Only three main studies reported in [19,20,21] have been carried out which investigate crumpled aluminium foil as a comparative material to other cellular materials. Bouaziz et al. [19] compared their results of crumpled aluminium foil to commercial metal foams and found they may have similar potential as a low cost alternative in some instances due to some shared characteristics. These studies show a relationship between increasing relative density ($\rho_{r}$) and increasing compressive strength ($\sigma_{Y,E}$). The $\rho_{r}$ rather than other factors, is the fundamental variable in mechanical performance and energy absorbance of cellular materials [22].

**Figure 1:**
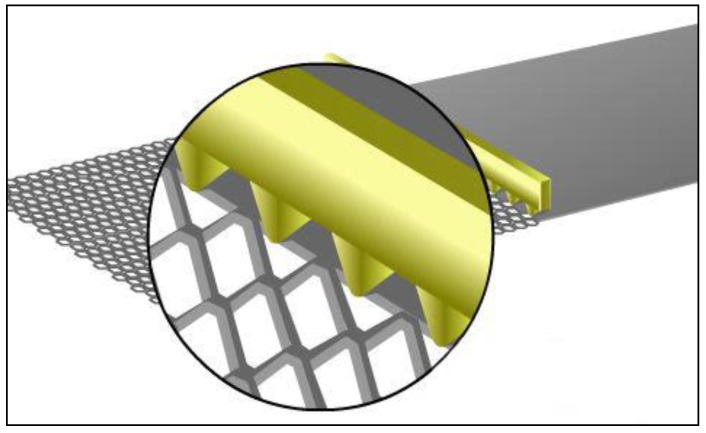
Expanded metal process.

Expanded aluminium foil mesh is a material made of an aluminium sheet which has been simultaneously sheared and expanded, creating a mesh structure. Figure 1, presents the expanded aluminium foil mesh. The process for expanding metal was first patented in Hartlepool, United Kingdom, in 1884, by John French Golding, the company exists to this day and provided samples for the current research. Expanded metal is a versatile material with potential for many applications, however, there has been no research investigating the mechanical properties of a crumpled expanded foil mesh. A review of expanded metal patents up to 2009 was published by Smith showing a wide range of applications [23]. Subsequent work has been performed to understand the compressive and impact behavior of expanded metal tubes [24,25] and nested tubes placed concentrically to form a 3D structure. Expanded metal tubes show improved energy absorbance [26,27] including with additional foams added [28]. Current research relating to expanded metal focuses predominantly on its application as a filter media [29] and its common structural applications [30,31]. Potential for applications in fuel cell technology has also been assessed [32].

The mesh foil when compressed presents an interesting alternative to studies on standard crumpled aluminium foil due to the differences in sheet geometry and resulting structure of the bulk material. The expansion process has effects on the material through work hardening originating from the shearing while the fully connected system allows the mesh to maintain strength [33,34]. Quantifying the yield strength and Young’s Modulus of the material at various densities is something most previous studies on crumpled foil did not do [19,20]. The compressed foil mesh has a purely interlocking structure by simply crumpling and compressing the material, creating a series of plastically bent vertices within the foil.

Much work has been presented on the compressive behavior of traditional aluminium metal foams and porous metals defining the static and dynamic behavior [35,36,37,38,39], energy absorbance [40] and the impact of pour density and structure [41,42,43,44]. Building on this previous work it is important to study the effect of $\rho_{r}$ on the compressive load response of crumpled aluminium foil mesh (CAFM) to quantify the mechanical properties and gain insight into its potential as a commercial cellular material. In addition, there is a need to study the structure of the CAFM using CT Tomography and compare the findings from the investigation of CAFM with crumpled aluminium foil sheet.

## 2. Methodology

The methodology adopted in this investigation is presented and discussed under three sub-headings. These are the materials and forging of the crumpled aluminium foil meshes (CAFMs), CT Tomography and uniaxial compression loading.

### 2.1. Materials and Forging of the CAFMs

**Figure 2:**
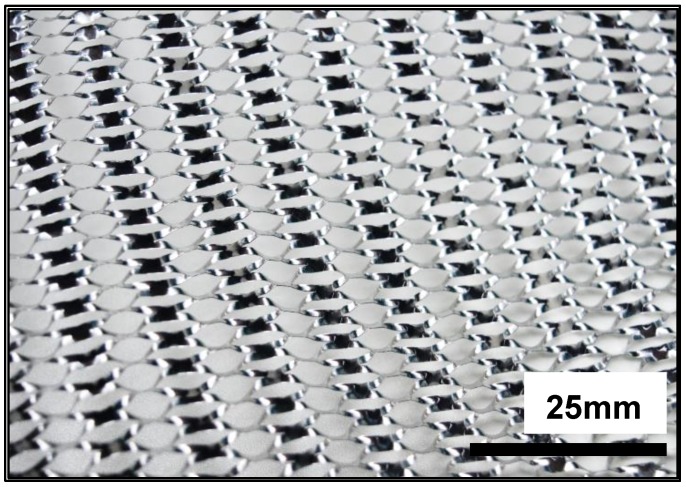
Expanded aluminium foil mesh.

The material used to manufacture the CAFMs is 0.05 mm thick 1200 H19 series aluminium sheet. The density of the aluminium is 2.7 g/cm^3^. The Expanded Metal Company, Hartlepool, UK, processed the mesh using bespoke machines. The mesh manufacturing process involves the use of shearing anvils which have 19.05 mm profile. On manufacture, the foil mesh produces an expanded diamond foil pattern. Figure 2 depicts the expanded aluminium foil mesh (EAFM).

**Figure 3:**
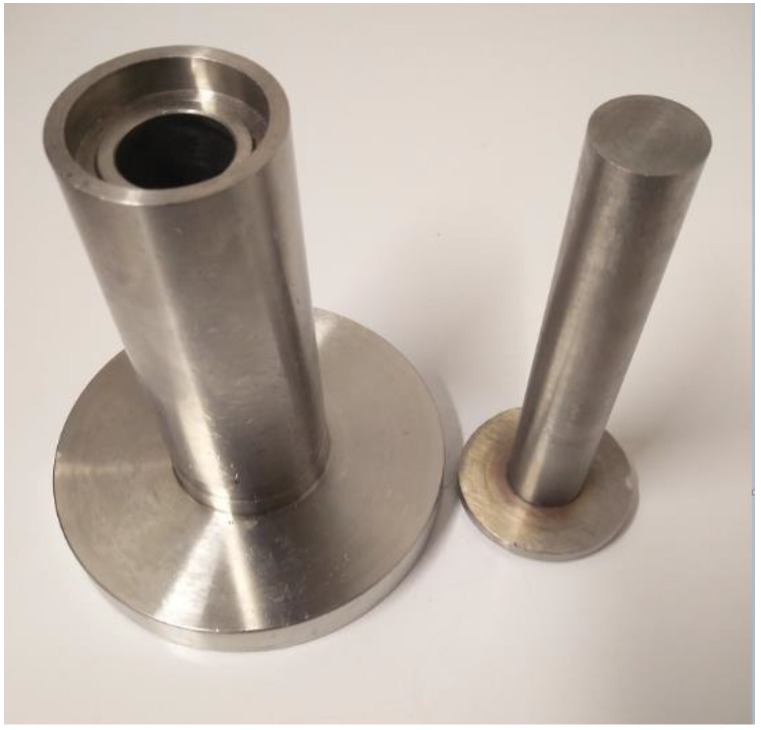
A punch and die tooling system.

**Figure 4:**
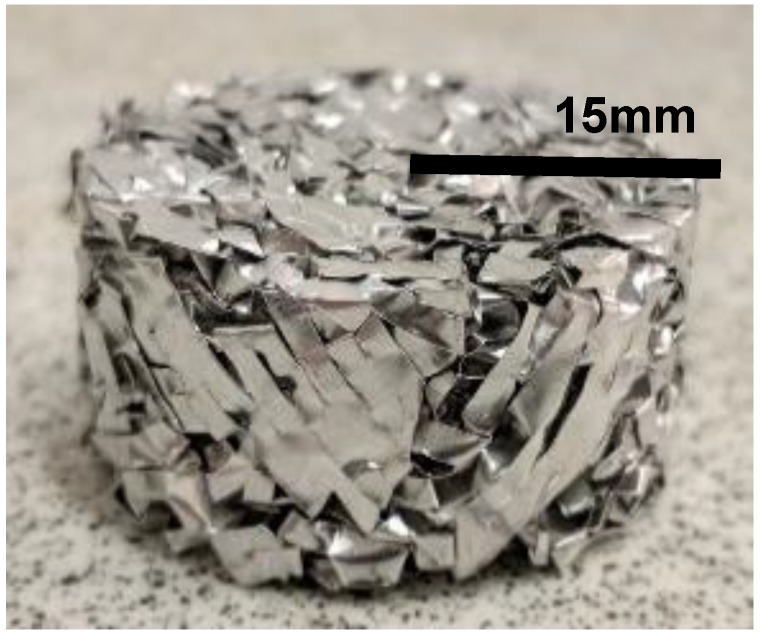
A crumpled aluminium foil mesh.

A CAFM is forged by manually hand crumpling a 200 × 200 mm dimension EAFM into a ball known as crumbled mesh ball (CMB) in this study. The CMB is introduced into a cylindrical punch and die tooling system. The die tooling is shown in Figure 3. It consists of two components. A punch which slides into a stationary die. The relative motion of the punch towards the stationary die compresses the ball to a bulk material of desired density by cold forging. The resultant material is called crumpled aluminium foil mesh (CAFM) in this research. Figure 4 depicts a CAFM. The magnitude of the displacement of the punch and thus the final height of the die, determines the density of the CAFM. The die is 15 mm in radius r and has a variable height h which allows the punch to slide in it.

**Figure 5:**
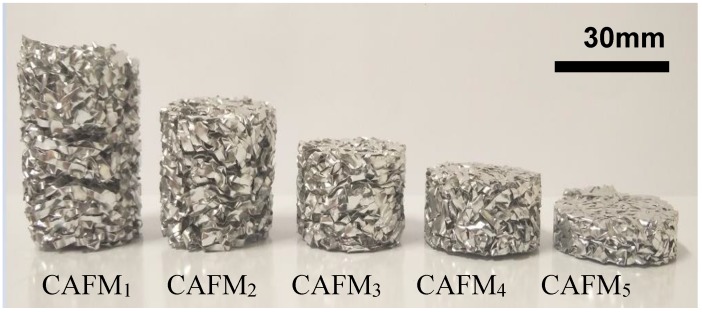
The five crumpled aluminium foil meshes (CAFMs).

Appropriate forces, at a displacement rate of 4 mm/min, are applied on the punch to compress five CMBs to five CAFMs of different densities. Figure 5 presents the five CAFMs. The relative density of each CAFM, $\rho_{r_{i}}$ may be defined as the ratio of the density of the CAFM, $\rho_{\mathrm{CAFM}}$ to that of solid aluminium, $\rho_{s}$. Mathematically, it is expressed:

(1) $$\rho_{r_{i}}=\frac{\rho_{{\mathrm{CAFM}}_{i}}}{\rho_{s}}=\frac{m_{\mathrm{CAFM}}}{{\pi r}^{2}{\times h}_{i}\times \rho_{s}}$$

where $\rho_{{\mathrm{CAFM}}_{i}}$ and $h_{i}$ are the densities and heights of an individual CAMF, $m_{\mathrm{CAFM}}$ is the mass of the CAFM which is a constant. The i=1, 2, 3, 4, 5 is a representative of individual CAFM.

Each CAFM creation is repeated 10 times to ensure the process is stable and easily reproducible. The $\rho_{r}$ standard error of the mean is designated as $SE\bar{\rho_{r}}$ and computed. The height and mass of each CAFM varied within 1 mm and 0.2 g, respectively. The practice ensures that the maximum difference between identical samples $\rho_{r}$ is only 0.001. Table 1 depicts the properties and details of the CAFMs forged from the compression process.

### 2.2. Computed Tomography 

The internal structure of the forged CAFMs detailed in Section 2.1 are studied. To carry out the study, X-ray computed micro-tomography (X-ray μCT) is used to image the internal structure. It is a non-destructive technique used to examine the internal structure of opaque objects. Researchers including [45,46,47] have successfully employed the technology in their research of similar nature.

An X-ray μCT machine has three main components. These are an x-ray source, rotary table and x-ray detector. The machine working principle is founded on measurement based on Beer-Lambert law. The measurement is performed by collecting hundreds of 2D projections (often called radiograms) using a flat panel detector while the sample is rotated 360 (or 180) degrees. The projections are then used to generate 3D images using reconstruction algorithms [48]. The images can be generated both using bench-top scanners and synchrotron facilities [49]. The 3D images are 3D maps of the density of the specimen.

In this work, the X-ray μCT imaging is utilised to quantitatively analyse the internal structure of the CAFMs non-destructively. The μCT scanner used is located at Durham University. It is an XRadia Versa-410 machine (Carl Zeiss AG, Oberkochen, Germany). In operation, the X-ray source voltage is 100 keV and all tomographic data is set at 10.7 μm voxel resolution. It scans the CAFM and collects 3D volume comprising of 3201 radiograms during a full 360° rotation of the text vehicle. Image quantifications are performed using Avizo (version 2018.2, Thermo Fisher Scientific, Waltham, MA, USA) and ImageJ (version 1.52f, NIH, Bethesda, MD, USA) software packages.

**Figure 6:**
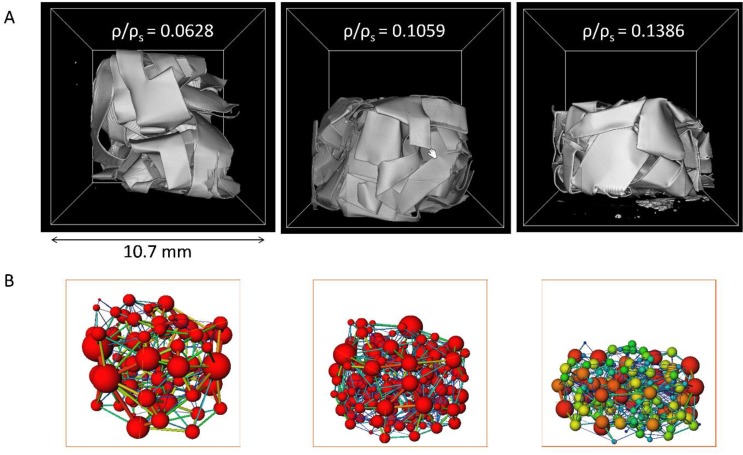
(**A**) The 3D rendering of the crumpled foil samples, (**B**) The pore-network models extracted from the images in (A).

Raw data obtained from the imagining is segmented using simple thresholding [50] such that the foil relative density matches the known density of each CAFM. To quantitatively assess the pore structure of these test vehicles pore-network models are extracted from the images. Figure 6A shows the 3D renderings of the scanned samples while Figure 6B depicts the obtained corresponding pore-network models for the images shown in Figure 6A. The network is composed of pores and pore-throats which are shown using balls and sticks. 

### 2.3. Uniaxial Compression Loading

The overarching aim of the study is to determine the compressive load response of the CAFMs as a function of $\rho_{r}$. The uniaxial compression loading of the CAFMs is carried out using an Instron 3367 tensile test machine (Instron Corp. MA, USA) utilizing a 30 kN load cell. Both open and close die techniques are employed in the loading. The relative motion of the punch towards the stationary die compressively loads the CAFM axially. The top flat platen of the Instron 3367 tensile test machine gives the punch a displacement rate of 4 mm/min. The magnitude of the applied force and the deformation of the CAFM is recorded by a computer attached to the machine. All samples are repeated 5 times to ensure test consistency. To further explore the elasto-plasticity of the CAFM’s the compressive loading was cycled several times at a range of stress limits (0.1–1 MPa). This data is specifically used to more clearly understand the real elastic region for the definition of elastic modulus (E).

#### Uniaxial Open and Close Die Compression Loading

**Figure 7:**
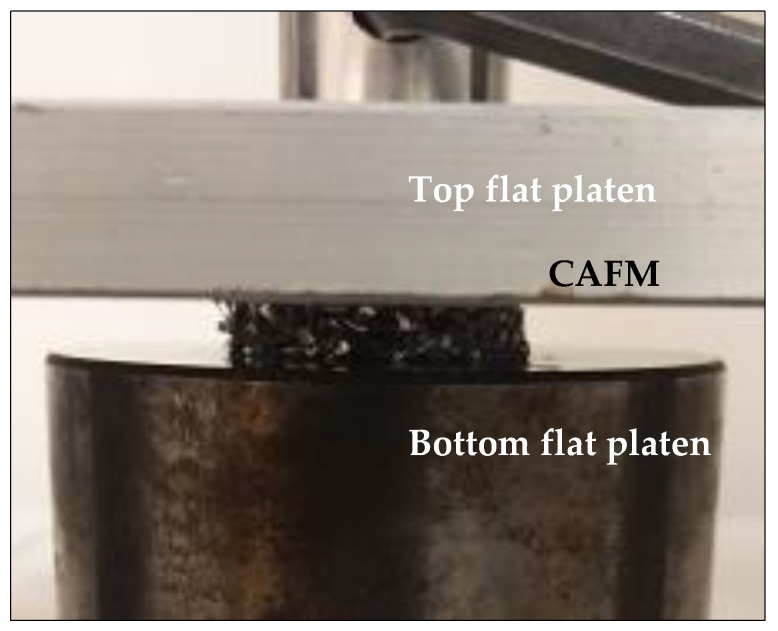
Open die compression loading showing the experimental set up and location of the CAFM sample.

In open die loading, each CAFM is sandwiched between top and bottom flat platens as shown in Figure 7. The Figure 7 presents the set-up of the experiment showing the details of the part including the top and bottom platen and a sandwiched CAFM. In close die loading, each CAFM is re-introduced into the punch and die tooling system as shown in Figure 3. The assembly—punch and die tooling system containing the CAFM is loaded in the universal test machine between the platens and forces applied on it through the displacement of the top flat platen.

To define the load response of each CAFM, its stress-strain relation is determined using the basic Equation (2):

(2) $$\sigma_{i}{=E\varepsilon }_{i},$$

where $\sigma_{i}$ and $\varepsilon_{i}$ are the compressive axial normal stresses and strains on each CAFM due to the loading. The magnitude of $\sigma_{i}$ is calculated by evaluating the ratio of the applied force, $F_{i}$ over the cross-sectional area of each CAFM. The mathematical expression is given by Equation (3):

(3) $$\sigma_{i}=\frac{F_{i}}{{\pi r}^{2}}.$$

**Figure 8:**
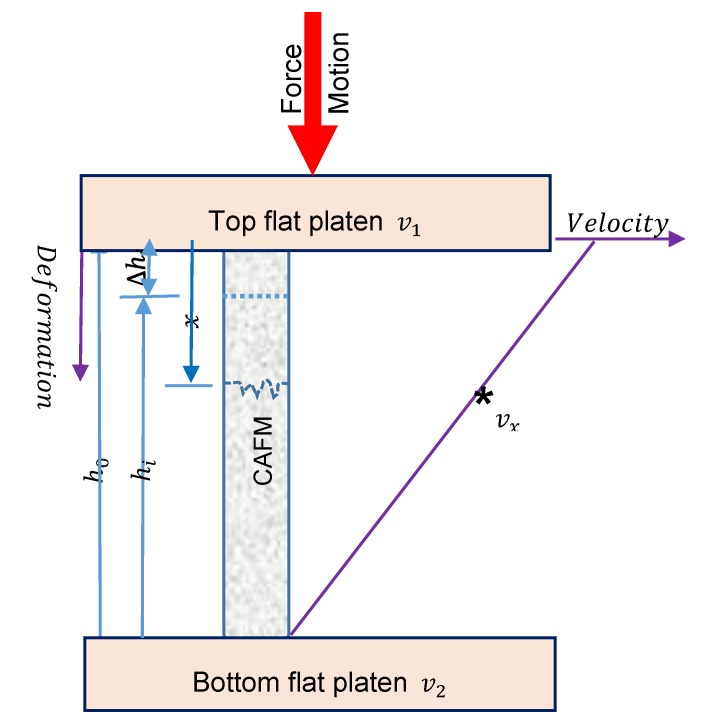
Diagrammatic representation of the loading mechanics.

The magnitude of $\varepsilon_{i}$ experienced by each CAFM is determined by considering its true (natural) strain under the loading. This concept is used to evaluate the degree of the deformation on the CAFMs considering that large deformation is encountered during the loading. It also takes into account the continuous and non-uniform variation in the deformation of the CAFMs during loading. The true strain otherwise known as logarithmic axial strain ($\varepsilon_{i}$) may be calculated from the diagrammatic representation of the loading mechanism presented in Figure 8. Considering Figure 8, the top and bottom platens have velocities $v_{1}$ and $v_{2}$ respectively. Each CAFM has an initial height of $h_{o}$ and a final height of $h_{i}$ while the relationship between the parameters and the change in height $∆h_{i}$ is described by Equation (4):

(4) $$h_{i}=h_{o}-∆h_{i}.$$

On loading, particles on a CAFM in contact with the top platen will have a velocity $v_{1}$ while those in contact with the bottom platen have a velocity $v_{2}$ Given that the velocity variation in the particles of the CAFM between the two platens is linear, a particle at a distance x from the top platen will have a velocity $v_{x}$ and strain rate $\frac{\partial \varepsilon_{x}}{\partial t}$ defined by Equations (5) and (6), respectively:

(5) $$v_{x}=\left(\frac{v_{2}{-v}_{1}}{h_{0}}\right){x+v}_{1},$$

(6) $$\frac{\partial \varepsilon_{x}}{\partial t}=\frac{\partial v_{x}}{\partial x}.$$

Combining Equations (5) and (6), obtain an expression for strain $\varepsilon_{i}$ on the CAFMs:

(7) $$\varepsilon_{i}=\int \left\{\frac{\partial }{\mathrm{dx}}\left[\left(\frac{v_{2}{-v}_{1}}{h_{0}}\right){x+v}_{1}\right]\right\}\partial t=\int \left\{\frac{v_{2}{-v}_{1}}{h_{0}}\right\}\partial t=\int \left\{\frac{\partial v\partial t}{h_{0}}\right\}.$$

From dynamics, the displacement deformation of a CAFM can be expressed as in Equation (8):

(8) $$\partial h_{0}=\partial v\partial t.$$

Thus, Equation (7) reduces to:

(9) $$\varepsilon_{i}=\overset{h_{i}}{\underset{h_{0}}{\int }}\;\frac{1}{h_{0}}\partial L_{0}=\ln\frac{h_{i}}{h_{0}}.$$

Substituting Equation (4) into Equation (9), $\varepsilon_{i}$ may be re-defined as in Equation (10):

(10) $$\varepsilon_{i}=\ln(1-\frac{∆h_{i}}{h_{o}}).$$

If the engineering strain for each CAFMs is designated as $\in_{i}$ and defined as in Equation (11):

(11) $$\varepsilon_{i}=\frac{∆h_{i}}{h_{o}}.$$

then the relationship between the two quantities is expressed thus:

(12) $$\varepsilon_{i}{=\ln(1-\varepsilon }_{i}).$$

Substituting Equation (10) into Equation (2), obtain Equation (13):

(13) $$\sigma_{i}=\mathrm{Eln}(1-\frac{∆h_{i}}{h_{o}}).$$

The Equation (13) is the analytical expression which models the response of each CAFM to the applied compressive uniaxial loads. 

The responses of the CAFMs loaded in both open and close die conditions are compared. Other strain rates are tested but seen not to effect test vehicle response under loading.

## 3. Results and Discussion

The results and their discussion are presented in six sub-headings. These are the CT tomography, effect of relative density on: load response, Young’s modulus, yield strength as well as the effect of compression method on compressive strength.

### 3.1. The X-ray Computed Micro-Tomography

**Figure 9:**
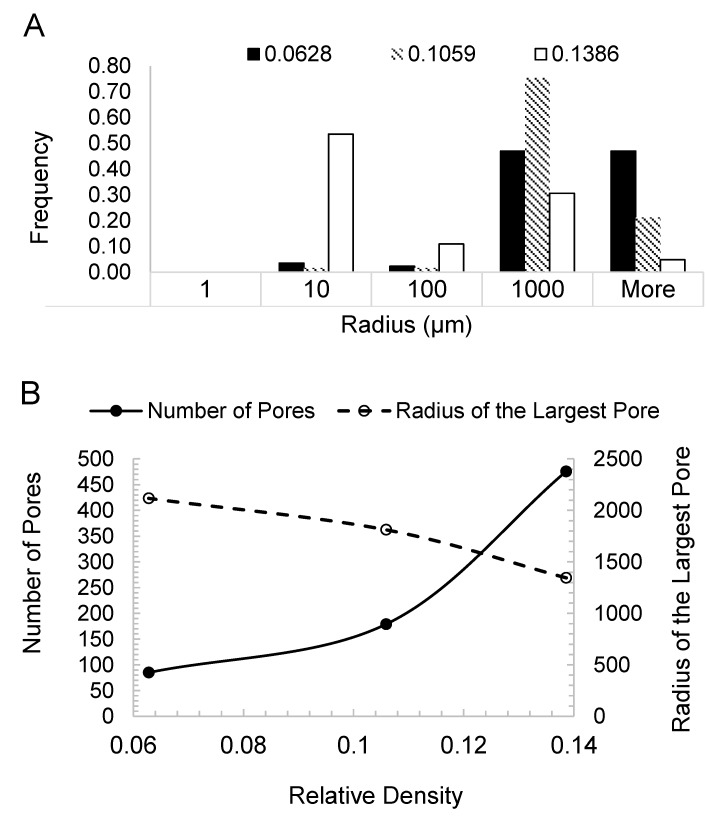
Computed tomography data on the three networks shown in Figure 6B. (**A**) Bar char representation of pore radius (μm) frequency, (**B**) Plot of number of pores and largest pore-size (μm) against relative density.

The data from the CT tomography for three relative densities 0.0628, 0.1059 and 0.1386 is presented in Figure 9. Figure 9A statistically presents the frequency of pore radius for each relative density. This is the pore-size distribution. The distribution shows that a denser CAFM has tighter pore-spaces and smaller pore sizes. Figure 9B presents the plots of number of pores and the radius of large pores against the relative density. It can be seen that CAFMs with higher relative density have a greater number of pores. It also shows that the radius of the largest pores decreases in denser CAFMs. These observations confirm that as compression progresses the number of pores in the cellular bulks increases while the radius of the largest pore decreases. The research reported in [20] recorded similar behavior when foils are crushed. This analysis quantitatively measures the impact of compaction on the pore structure of the CAFMs. The X-ray computed micro-tomography suffices to model the internal structure of the CAFMs and thus cellular condensed bulk matter for macro-structural research.

### 3.2. Effect of Relative Density on CAFMs Compressive Load Response

**Figure 10:**
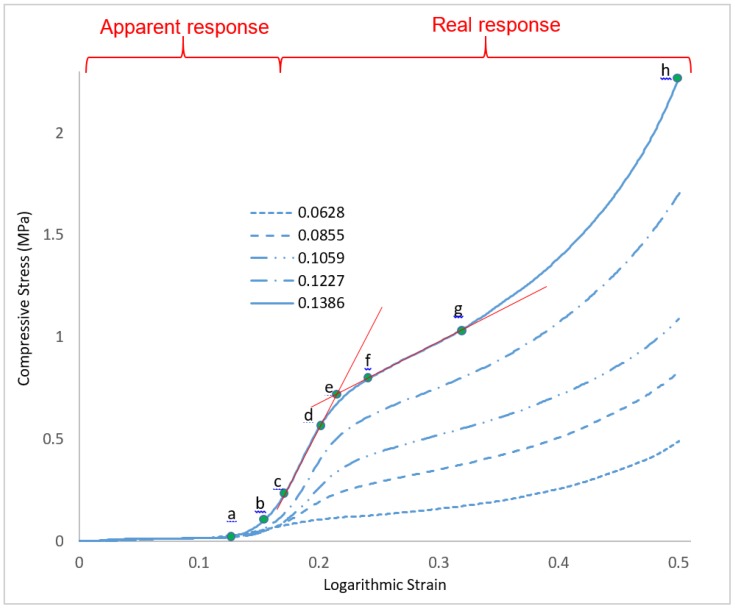
Stress-strain response of CAFMs under open die uniaxial compression loading at 4 mm/min punch velocity. The critical compressive stages in relation to the logarithmic strain are identified with the green dots on the response profile. Point *0-c* marks the apparent response while the point *c-h* is the real response. The latter includes the elastic response in region *c-d*.

The stress-strain plots of the compressive stresses against the logarithmic strains of the CAFMs are presented in Figure 10. The plots demonstrate identical response profile irrespective of the relative density ($\rho_{r})$ of the CAFM. They are characterised by two distinctive responses. An initial apparent response designated by region *0c* and a real response designated by region *ch* in each of the CAFM’s profile.

The apparent response region 0c consists of three stages. These are the: apparent proportional region 0a, apparent elastic limit b and the apparent yield point c in each profile. For each of the profiles, in region 0a, the CAFMs are assumed to have the same magnitude of ${d\sigma }_{i}/{d\varepsilon }_{i}$ which is constant and about 0.144 MPa in magnitude. The assumption is because a very small magnitude of stress is involved in this region. In addition, a holistic view of the entire load response profile as shown in Figure 10 depicts the region as linear and proportional. The response is seen as a step function which demonstrates early perfectly plastic response ability of the CAFMs within its proportional elastic region. A similar observation is reported by Cottrino et al. who stated in their research that within the elastic region of their foil it was likely that there was plastic deformation on a very small scale in the interlocked vertices of the structure [20]. It is inferred that during the constant compressive stress under continuous loading, the applied loads are not borne by the CAFMs materials, they are used to tighten the pores spaces. The CAFMs interlock through their many vertices during plastic deformation. The apparent elastic limit and the yield point of the CAFMs varies with $\rho_{r}$. Denser CAFMs demonstrate a higher apparent elastic limit and yield point. The apparent load response region is caused by the expanded nature of the foil occasioned by it being a mesh. The expanded mesh nature provides a potential for the CAFMs to trade off stress absorption with increase in strain and $\rho_{r}$. This nature supports the CAFMs to tighten pore spaces leading to further interlocking at the cellular level. This large deformation has a limiting value at point *c* where the CAFMs begin to demonstrate possession of properties similar to crumpled metal foil. The knowledge from this observation will be useful when designing sensitive energy absorption systems of the order of 0.25 MPa.

The real response region *ch* consists of three stages. These are the: real proportional region cd, real elastic limit *f* and the real yield point *g*. The point *c* is the onset of the densification stage where the CAFMs’ load response approximate that of crumpled metal foils. In region cd, the CAFMs have constant ${d\sigma }_{i}/{d\varepsilon }_{r}$ which varies with the $\rho_{r}$. In comparison to the region *0a*, the region has much higher magnitude of ${d\sigma }_{i}/{d\varepsilon }_{r}$. The CAFM at the region possesses higher $\rho_{r}$ and are thus stiffer. The macroscopic deformation at the cellular level is diminished as the pores becomes relatively incompressible owing to the interlocked vertices. The CAFMs start to behave as a continuum under further loading. Similar observations are reported by the [19,21]. This is unlike the behavior of metal foams that show a long plateau after the yield stress before eventually reaching the densification strain [4]. The cd region is used to compute the real Young’s Modulus of the CAFMs. Detailed discussion on the computation is presented in Section 3.3. The real elastic limit and the yield point of the CAFMs are observed to vary with the $\rho_{r}$. The denser the CAFM the higher the magnitude of the real elastic limit and yield point. However, the point *e* is used to determine the effective yield point of the CAFMs. Detailed discussion on the determination and effect of the effective yield point are presented in Section 3.4.

**Figure 11:**
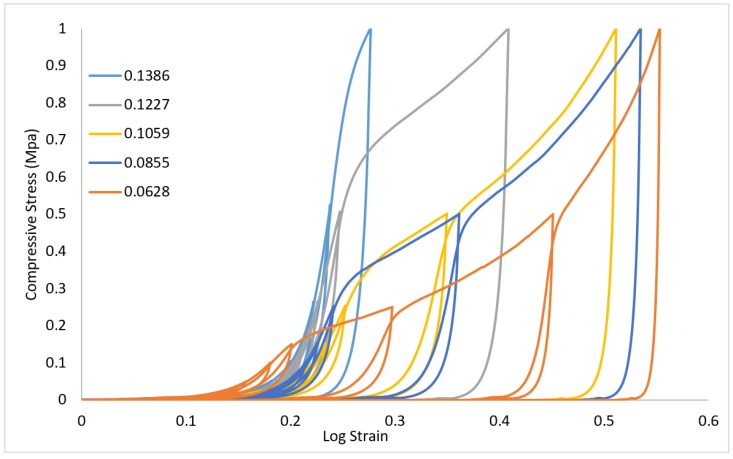
Consecutive loading–unloading stress–logarithmic strain curves of CAFMs under open die uniaxial compression loading.

**Figure 12:**
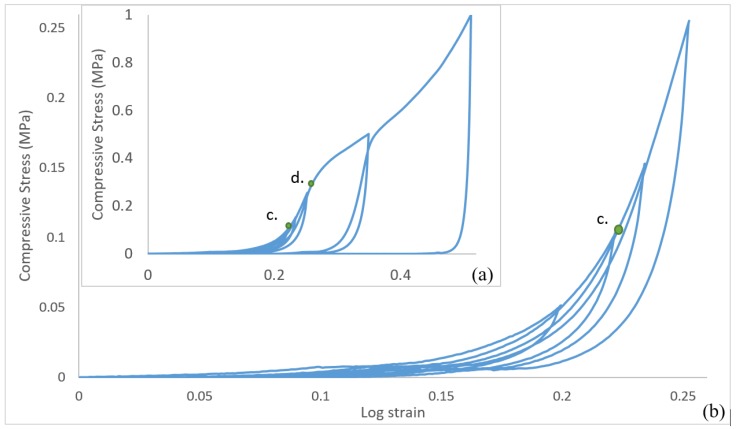
Loading–unloading of CAFM with $\rho_{r}$ 0.1059 showing linear elastic cd region: (**a**) full loading-unloading graph and (**b**) amplified initial part of the curve. The elastic region, defined as points *c-d*, is annotated to the curves.

In order to understand the elasto-plasticity of the CAMF’s, loading–unloading cycles are applied across a range of strains. Figure 11 presents the results of these loading-unloading cycles on the CAFM’s. Figure 12 provides an isolated plot of the load response of CAFM_0.1059_ to visualize the elastic cycling at low strain. Within the region 0–d the loading–unloading curves display a very small hysteresis between the loading and unloading cycles. Below point *d* the material response is primarily elastic. The plot demonstrates that insignificant energy losses occur as compression and interlocking progresses but the material response remains predominantly elastic in the region c–d. Once interlocking is significantly achieved in the region 0–c the CAFM approximate to a continuum and responds similarly to bulk aluminium materials.

The CAFMs demonstrate characteristics of both porous metals and entangled materials as they display strain hardening immediately after the yield stress. The relationship between increased $\rho_{r}$ and increased compressive strength is linked to the tightening of the pore-spaces leading to an increase in interlocking of the foil mesh. These findings are in line with the behavior of cellular materials reported in previous studies [19,20,21]. The knowledge of the real response of the CAFMs will be useful in designing energy absorption systems of the order greater than 0.25 MPa.

### 3.3. Effect of Relative Density on the CAFMs Real Young’s Modulus $E_{R}$ (Stiffness)

**Figure 13:**
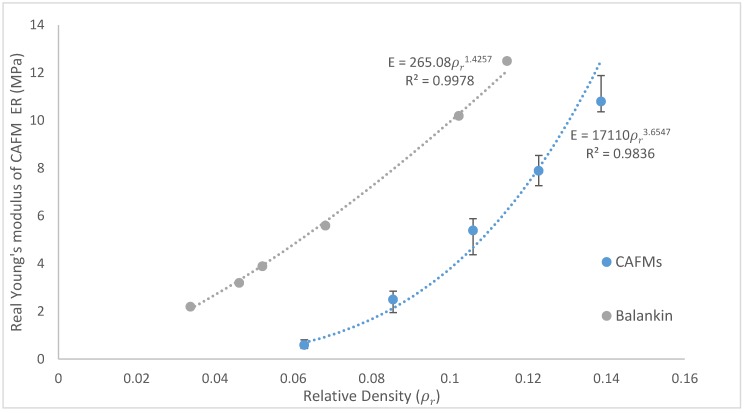
Plot of real Young’s modulus against relative density of CAFMs and [21].

The real Young’s Moduli ($E_{Rs}$) of the CAFMs is calculated using the gradient of the real region cd shown in Figure 10. The gradients of the region cd under open die uniaxial compression loading are found to be 0.0628 (σ = 0.6287ε − 0.0334), 0.0855 (σ = 2.4701ε − 0.308), 0.1059 (σ = 5.5358ε − 0.826), 0.1227 (σ = 7.9493ε − 1.381), 0.1386 (σ = 10.778ε − 1.6101). A plot of the $E_{Rs}$ against $\rho_{rs}$ of the CAFMs is presented in Figure 13. It can be seen from the plot that the CAFM with the highest $\rho_{r}$ of 0.1386 (${\mathrm{CAFM}}_{0.1386}$) has the highest $E_{R}$ of 10.78 MPa while the CAFM with the lowest $\rho_{r}$ of 0.0628 (${\mathrm{CAFM}}_{0.0628}$) has the lowest $E_{R}$ of 0.6287 MPa. The current study is named CAFMs. The figure also shows the plot of similar research study reported by [21] who used the same region in computing the $E_{R}$ of the crumpled metal foil in their investigation. The plot of the current investigation demonstrates that the relationship between the $E_{R}$ and the $\rho_{r}$ is a power function of the form $E_{R,}{=17110\rho }_{r}{}^{3.6547}$ (R^2^ = 0.9836). A power law is used to fit the points because it yields a zero magnitude of $E_{R}$ at zero $\rho_{r}.$ The significance of this is that at zero $\rho_{r}$ the $\rho_{CAFMs}$ is zero and a space under consideration does not contain a CAFM. The regression line is a close fit which allows for good $E_{R,}$ prediction of CAFM for untested $\rho_{r}$. Previous studies use a power law fit to represent the relationship between $E_{R}$ and $\rho_{r}$ [21]. A comparison of the two plots shows identical characteristics. The results of the current study show a significant lower magnitude of $E_{R,}$ for comparable $\rho_{r}$. The observation is expected because the CAFMs are made of expanded metal foil mesh which ought to demonstrate more plasticity and flow behaviour compared to non-expanded metal foil used by [21]. Based on the generated results, CAFMs may have applications in the design of energy absorption systems where less stiffness is required for the same $\rho_{r}$

### 3.4. Effect of Relative Density on the CAFMs Effective Compressive Yield Strength $\sigma_{Y,E}$

The effective compressive yield strength ($\sigma_{Y,E}$) of the CAFMs are at the point *e* in each CAFM’s compressive stress against logarithmic strain plot shown in Figure 10. The point *e* is the intersection of two lines. One of the lines is tangent to the proportional region cd while the other line is tangent to both real elastic limit *f* and real yield point *g*. The two tangents and their intersection are shown in Figure 10. Similar approach to the determination of $\sigma_{Y,E}$ is reported by Balankin et al. [21].

**Figure 14:**
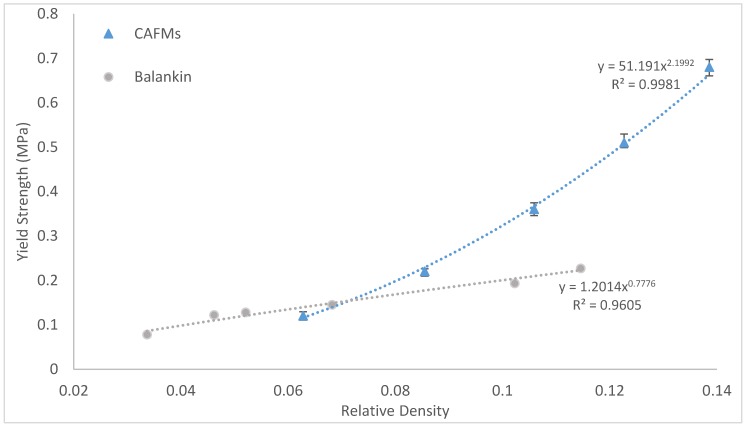
Plot of real compressive yield strength (MPa) against relative density of CAFMs and [21].

A plot of the CAFMs $\sigma_{Y,E}$ (extracted from Figure 10) against the $\rho_{r}$ is shown in Figure 14. The current study is named CAFMs. The figure also shows the plot of similar research study reported by Balankin et al. [21] who used the same approach in the determination of $\sigma_{Y,E}$ of crumpled metal foil in their investigation. The plot of the current investigation demonstrates that the relationship between the $\sigma_{Y,E}$ and the $\rho_{r}$ is a power function of the form $\sigma_{Y,E}={51.191\rho }_{r}{}^{2.1992}$ (R^2^ = 0.9981). A power law is used to fit the point because it yields a zero magnitude of $\sigma_{Y,E}$ at zero $\rho_{r}.$ The significance of this is that at zero $\rho_{r}$ the $\rho_{CAFMs}$ is zero and a space under consideration does not contain a CAFM. The regression line is a close fit which provides a good $\sigma_{Y,E}$ prediction of CAFMs for untried $\rho_{r}$ A comparison of the two plots shows identical characteristics of power law fits. The results of the current study show a significantly higher magnitude of $\sigma_{Y,E}$ for values of $\rho_{r}$ greater than 0.065 and a lower magnitude for $\rho_{r}$ less than 0.065. The higher magnitude of $\sigma_{Y,E}$ recorded by the CAFMs occur because the expansion process used in making the mesh had pre-work hardened the material and provided more structure for tighter compaction compared to the standard metal foil sheet used by the authors of [21].

Based on the generated results, CAFMs can be more useful in the design of energy absorption systems where higher yield strength is required for $\rho_{r}$ greater than 0.065.

### 3.5. Effect of Compression Technique on CAFMs Real Compressive Strength $\sigma_{Y,R}$

**Figure 15:**
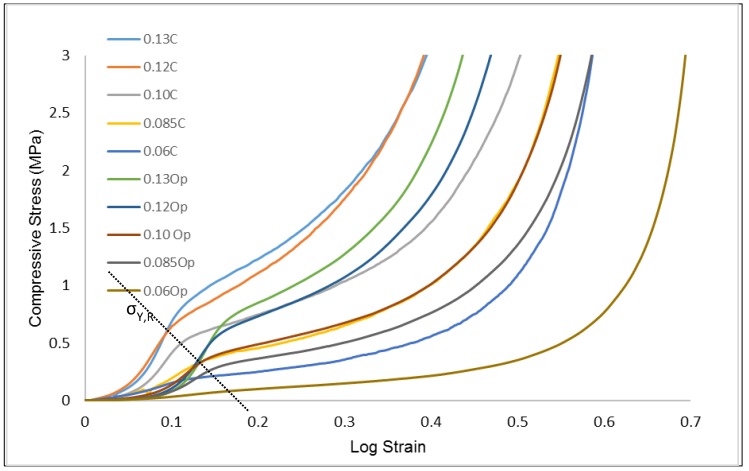
Stress–strain plots for open and closed die compression loading of the CAFMs.

The effect of open and closed die compression loadings on CAFMs real compressive strength is studied. Figure 15 presents the plots of compressive stress against the log strain of both techniques for the CAFMs. The plots are depicted on the same figure for easy comparison of the values. The $\sigma_{Y,R}$ of each CAFM is determined as explained in Section 3.2 and indicated in Figure 15. It can be seen from the plot that the $\sigma_{Y,R}$ of CAFMs loaded in closed die technique is higher than the ones loaded in open die compression. The higher magnitude is due to perfectly uniaxial compression caused by the die wall limiting radial expansion of the CAFMs during loading. In addition, the die cylindrical wall introduces frictional force which increases the load from the punch. These results are in agreement with previous studies [20,21] on aluminium foils. The authors reported the existence of friction and therefore higher compressive strength when the material is compressed within a die.

## 4. Conclusions

The compressive load response of aluminium foil mesh crumpled into cylindrical bulk matter of several relative densities, $\rho_{rs}$ is studied to provide new knowledge on the effect of $\rho_{r}$ on its load bearing capacity. Based on the results generated from the investigation it can be concluded that the uniaxial compressive load response of the CAFMs is dependent of their $\rho_{rs}$. In specific terms, the real Young’s modulus $E_{R}$ and the effective compressive yield strength, $\sigma_{Y,E}$ are significantly dependent on its relative density. The real Young’s modulus $E_{R}$ increases with increase in $\rho_{r}$. Thus, denser CAFM is stiffer than the less dense ones. It is concluded that the higher the $\rho_{r}$ of CAFM, the greater the $\sigma_{Y,E}$ and thus the more is the load bearing capacity. The relationship between the $E_{R}$ $\sigma_{Y,E}$ and $\rho_{r}$ can be modelled using the power law. The compression techniques (open or closed die) employed in the loading of the CAFM determine the magnitude of the $E_{R}$ and $\sigma_{Y,E}$.

This knowledge can be used to evaluate the application of crumpled aluminium foil mesh (CAFM) technology as an alternative to traditional porous metal and identify novel applications of the material.

## Figures and Tables

**Table 1:** Properties and details of the crumpled aluminium foil meshes (CAFMs).

S/No	Crumpled Aluminium Foil Mesh (CAFM)	Mesh Dimension (mm^2^)	h_o_ (mm)	ρr=ρρs	$SE\bar{\rho_{r}}\;\left(\times 10^{-5}\right)$
1	${\mathrm{CAFM}}_{1}$	200 × 200	31.57	0.0628	15.212
2	${\mathrm{CAFM}}_{2}$		22.59	0.0855	21.758
3	${\mathrm{CAFM}}_{3}$		17.45	0.1059	13.958
4	${\mathrm{CAFM}}_{4}$		15.20	0.1227	16.786
5	${\mathrm{CAFM}}_{5}$		14.15	0.1386	13.908

## Data Availability

The raw data required to reproduce these findings are available to download from [http://dx.doi.org/10.17632/tjxwj2r7s7.1]. The processed data required to reproduce these findings are available to download from http://dx.doi.org/10.17632/tjxwj2r7s7.1.
